# Target, Suspect and Non-Target Screening of Silylated Derivatives of Polar Compounds Based on Single Ion Monitoring GC-MS

**DOI:** 10.3390/ijerph16204022

**Published:** 2019-10-21

**Authors:** Bhekumuzi Prince Gumbi, Brenda Moodley, Grace Birungi, Patrick Gathura Ndungu

**Affiliations:** 1 School of Chemistry and Physics, University of KwaZulu-Natal, Durban 4000, South Africa; GumbiB@ukzn.ac.za (B.P.G.); Moodleyb3@ukzn.ac.za (B.M.); 2 Department of Chemistry, Mbarara University of Science and Technology, Mbarara 1410, Uganda; gbirungi@must.ac.ug; 3 Department of Chemical Sciences, University of Johannesburg, Johannesburg 2028, South Africa

**Keywords:** suspect and non-target analysis, GC-MS, emerging pollutants, pharmaceutical and personal care products

## Abstract

There is growing interest in determining the unidentified peaks within a sample spectra besides the analytes of interest. Availability of reference standards and hyphenated instruments has been a key and limiting factor in the rapid determination of emerging pollutants in the environment. In this work, polar compounds were silylated and analyzed with gas chromatography mass spectrometry (GC-MS) to determine the abundant fragments within the single ion monitoring (SIM) mode and methodology. Detection limits and recoveries of the compounds were established in river water, wastewater, biosolid and sediment matrices. Then, specific types of polar compounds that are classified as emerging contaminants, pharmaceuticals and personal care products, in the environment were targeted in the Mgeni and Msunduzi Rivers. We also performed suspect and non-target analysis screening to identify several other polar compounds in these rivers. A total of 12 compounds were quantified out of approximately 50 detected emerging contaminants in the Mgeni and Msunduzi Rivers. This study is significant for Africa, where the studies of emerging contaminants are limited and not usually prioritized.

## 1. Introduction

Many polar micro-contaminants such as illicit drugs, personal care products, plasticizers, pharmaceuticals and flame retardants exist in the environment, as evidenced from various studies [1,2,3,4,5]. Additionally, several transformation products can be formed from these various micro-contaminants, of which just a few have been identified [6,7,8]. Transformation products may be toxic compared to the parent compound [9]. The level of toxicity maybe further exacerbated by the presence of potentially harmful unknown compounds that are simultaneously present in the environment together with priority contaminants. Methods on how to account for these various micro-contaminants and include such compounds in the analysis of environmental samples is of growing interest. Moreover, the existence of organic matter such as humic acid can obstruct the pre-concentration of analytes, mass spectrometry ionization and determination of anthropogenic compounds.

In the literature, there are three approaches that are normally used for the analysis of compounds: target, suspect and non-target analytes [10,11,12]. Targeted methods are constrained by the availability of analytical standards, accompanied with costly reference standards, and therefore the identification of emerging contaminants within the environment may be delayed. Suspect screening has the advantage of using databases with known analyte structural properties and molecular ion formulae, which are computationally compared to mass spectrometry spectral data to give potential similarities to the compound of interest. The third approach, non-target analysis, is a growing focus but more challenging to carry out because no prior information is usually available [13,14,15]. The environmental samples could contain thousands of peaks [16,17]. As a result, steps need to be taken to decrease the amount of peaks to a manageable number, calculate suitable molecular formulae, determine the isotopic patterns, and perform defect analysis of the mass defect and time prediction of the retention time [14,18,19].

Based on its selectivity or accuracy, mass spectrometry is becoming the designated technique to detect and identify anonymous compounds in the environment [20]. The technique is based on correlation of the fragmentation pattern of mass spectra stored in the gas chromatography mass spectrometry (GC-MS) libraries. However, application of GC-MS in full-scan screening mode leads to low sensitivity/detection limits, poor selectivity and detection of many peaks in a sample, leading to false reporting of environmental data [21]. Moreover, most emerging pollutants are polar and GC-MS is not usually suitable for their analysis in environmental matrices. Recently, the development of precise high-resolution mass spectrometry (HRMS) opened a new paradigm in analytical processing or handling data for non-targeted compounds. Multi-residue analytical techniques are playing major roles to provide full information about overall environmental contamination, which can fast-track the identification of unknown compounds in the environment at large [10,19,22,23].

The combination of GC-MS, derivatization of samples and detection in single ion monitoring (SIM) mode may offer some advantages when compared to HRMS methods in terms of selectivity, precision, sensitivity, simplicity, cost and interference due to matrix effects. However, the lack of libraries for the derivatized compounds in the SIM mode compared to the full-scan mode has resulted in this method being overlooked for the screening of environmental samples in favor of HRMS. In general, the most abundant fragment ion results from fragmentation processes that form the most stable products, which determines the detectability of the analyte. The abundance of fragment ions of interest are affected by their stability. Silylated compounds produce informative and stable fragments, which enhances the detection limits of the GC-MS. In most situations, silylation reactions generate only the desired derivatives. However, the mass spectra of many silylated compounds may not be available in common mass spectral libraries. Other side reactions due to derivatization, such as alkylation and acylation, produce undesired compounds, and the use of Lewis acids as catalysts in aqueous solutions can affect the longevity of the column packing and decrease the instrument sensitivity. GC-MS methods are known to be cheap and robust if they are developed properly. These methods are needed in our technologically advanced societies, where approximately 100,000 chemical substances are used daily, with a large number of new compounds being discovered and registered every year [16]. Because of the potential negative environmental and/or health impact connected with contact to some of these chemicals, data regarding the presence of known and unknown compounds in the environment must be provided and regularly updated.

In this work, various pharmaceuticals and personal care products (PPCPs) were silylated, and abundant fragments were identified and used in single ion monitoring mode for quantification. As the publically available libraries/databases, such as National Institute of Standards and Technology (NIST), contain fragments obtained from scanning mode, an in-house database was built based on silylated abundant ions by injecting derivatized standards in the GC-MS. Then, quantitative and qualitative analysis of polar compounds that can be classified as emerging contaminants was carried out on samples from two rivers in South Africa using the single ion monitoring GC-MS method. In addition, suspect and non-target analyses were performed to identify some more contaminants in the environmental samples. NIST was used for searching and prediction of unknown compounds within samples. We were able to screen samples from portable water, wastewater, biosolid and sediment samples from the Mgeni and Msunduzi Rivers over a 1-year period.

## 2. Materials and Methods

Glassware and amber bottles were cleaned with phosphate-free soap bought from Dynachem (Durban, South Africa) and were left in the acid bath for a day. Then all glassware (Searle, Vervaardig, South Africa) were further washed with 5% dichloromethylsilane, finally rinsed with methanol, and then placed in the oven (Prolab, Durban, South Africa) at 60 °C for 12 h. Micro-volumes were transferred by a micropipette plus kit bought from Dragon lab (Beijing, China) ranging between 0.5 to 1000 μL. All glass fibre filters were purchased from Pall Corporation (Johannesburg, South Africa). Extraction cartridges (Oasis HLB 20 cc (1 g and 60 mg) LP, tC18 environmental cartridge sepak-pak and sepak-pak plus CN cartridge) were bought through Microsep (Durban, South Africa), a local supplier of Waters Corporation (Milford, MA, USA) products in South Africa. A Shimadzu QP2010 SE GC-MS (Kyoto, Japan) equipped with an autosampler and autoinjector (AOC-20i) (AOC-20s) was used for analysis. The capillary column, Crossbond 5% diphenyl/95% dimethyl polysiloxane (intercap 5 Sil MS 0.25 mmL. D χ 30 M df = 0.25 μm, non-polar) purchased from Restek (Bellefonte, PA, USA) was used for GC separation. Experiments were conducted at a room temperature of 20 °C.

### 2.1. Preparation of Stock Solutions

Working solutions of each standard surrogate standard cinnamic acid, 3-phenylprop-2-enoic acid internal standard (IS) 2-chlorobenzoic acid, and injector standard 4, 4-di-tert-butylbiphenyl (1000 μg/L) were dissolved in methanol and kept at 4 °C.

### 2.2. Sampling of Sediments and Water

**Figure 1:**
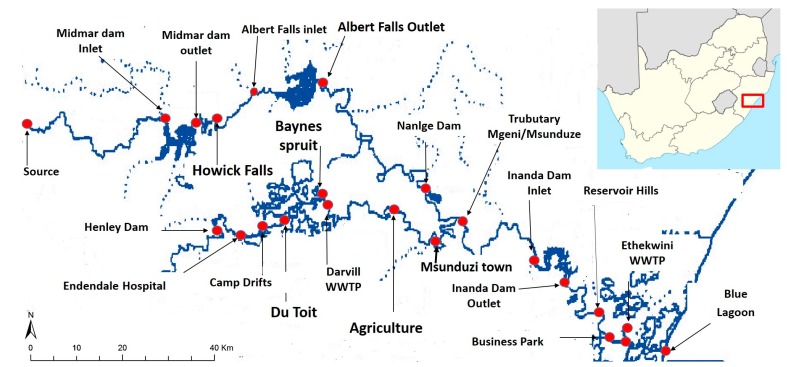
Sampling area of the Msunduzi and Mgeni Rivers in KwaZulu-Natal, South Africa. Grab samples were collected along all points indicated and analyzed using the developed method described in this work (map was drawn using GIS software (Caliper Corporation, Newton MA, United States of America) shape file).

The samples of river water and wastewater were collected in 2.5 L amber glass bottles. No preservatives were added, the samples were kept in a cooler box at 4–6 °C and transferred into the laboratory for further analysis. All sampling points were along the Msunduzi and Mgeni Rivers as shown in Figure 1.

Sediment samples were taken, along the riverbed (0–10 cm depth), from different sampling sites using the grab method and were covered with aluminum foil. Sediments were air dried at 30 °C for 3 days, then ground by hand in a ceramic mortar and pestle, and sieved through different layers of mesh to obtain a final particle size of 53 μm (600, 400, 300, 200, 100, 75 and 53 μm) to ensure consistency and normalization of the sample.

### 2.3. Sampling of Biosolids

Biosolid and wastewater samples were taken at the entrance of the wastewater treatment plants. The biosolid samples were partially concentrated solids separated from the sewage at wastewater treatment plants (WWTPs), as can be seen in Figure 1. These samples were dried under air and processed using the procedure described below to treat and extract analytes from sample matrices.

### 2.4. Sample Extraction

#### 2.4.1. Water

Solid phase extraction (SPE) of liquid samples was done using an Oasis HLB cartridge (Waters, Milford, MA, USA) (1 g) at pH 2 and pH 7; pH was controlled by adding 1 M of diluted sulphuric acid dropwise (pH changes were recorded by a pH meter). The Oasis HLB cartridges were successively preconditioned by additions of 6 mL methanol, and ultra-pure water (pH 2 or pH 7). Then 1 L of the sample was extracted/pre-concentrated using a flow rate between 5 to 10 mL min^−1^. After drying the cartridge in air for 30 min under a gentle stream of nitrogen, the sample was eluted with a total of 9 mL of extraction solution (acetone/ethyl acetate 1:1 6 mL; methanol 1 mL; acetonitrile 1 mL; 1 dichloromethane 1 mL).

#### 2.4.2. Sediments and Biosolids

Sediment samples were subjected to an ultrasound extraction procedure, and each sediment sample was treated twice. In a typical treatment step, a mass of 10 g of sediment per sample (based on dry weight) was placed in a centrifuge tube (50 mL) with 10 mL of solution (ethyl acetate/acetone (1:1) 8 mL and water/acetonitrile (1:1) 2 mL). The samples were initially shaken vigorously and then placed in an ultrasonic bath for 20 min at room temperature. In a centrifuge, the samples were subjected to a speed of 6000 rpm for 20 min and the content was decanted into a silylated glass bottle. After centrifugation, extracts from two-treatment steps (on one sediment sample) were mixed before the clean-up/SPE procedure.

The mixed extracts obtained in the ultrasound–centrifugation step were evaporated to 0.5 mL in a glass vial under a nitrogen stream. The contents of the vial were transferred into a 250 mL glass bottle via rinsing with 0.5 mL methanol, were made to 200 mL with Millipore water, and the pH was controlled to pH 2 or pH 7 with 1 M sulfuric acid. This solution was percolated through the Oasis HLB cartridge (60 mg), which was previously conditioned and eluted as described in Section 2.4.1.

#### 2.4.3. Recoveries and Procedural Blanks

Spiked sediments or biosolid samples were prepared by adding 10 μL solutions (10 mg/L) of the target analytes in ethyl acetate to an exact 10 g sample and the solvents were evaporated in darkness overnight under 15 °C to avoid degradation of the analytes by light. The concentration of analytes in the samples after drying was 10 ng/g. The blank samples were prepared to determine the absolute recoveries.

Spiked water samples were prepared by adding 100 μL solution (10 mg/L) of target analytes into 1 L of distilled water, river water or wastewater. The final concentration of target compounds in 1 L was 1 μg/L. Blank water samples were analyzed to determine absolute recoveries.

### 2.5. Derivatization

The extracts were re-dissolved into 100 μL of NIST + 1% TMCS, gently mixed while the vial was closed, and permitted to undergo a reaction at room temperature for 2 min. Then, the mixture was transferred into an oven to react for 30 min at 70 °C. After the derivatization process, the extracts were diluted up to 0.5 mL volume with acetonitrile and 2 μL of the derivatized sample extracted residue was injected into the GC-MS. Optimization of the derivatization procedure was done in our previously published work [24].

### 2.6. GC-MS Analysis

Selected suspect, target and non-target contaminants were detected by Shimadzu QP2010 SE equipped with auto-injector. At the beginning, the temperature of the column oven was kept at 70 °C, the injector-port temperature was maintained at 250 °C, and 2 μL sample extract residues were injected in the split-less mode. Helium was used as the carrier gas at a flow rate of 8.0 Ml/min and pressure of 61.5 KPa. The temperature was initially held at 70 °C for 1 min, then increased at a rate of 30 °C/min to 190 °C (kept for 1 min), then ramped at 15 °C/min to 230 °C (3 min) and lastly ramped at 30 °C/min to 270 °C, and then held for 1 min. The interface between the oven and ion source chamber was set at 200 °C, which was the same as the ion-source temperature. Filament electron energy was fixed at 70 eV. The ion trap detector (ITD) parameters were set as follows: scan mass range between 50–850 *m*/*z*, 4 min solvent cut-off and 30 min run-time. Single ion monitoring (SIM) mode was performed for detection of selected analytes. This GC-MS method was based on our initial work, and quantification of acidic drugs in surface waters [24].

### 2.7. Data Analysis (Including Software)

**Figure 2:**
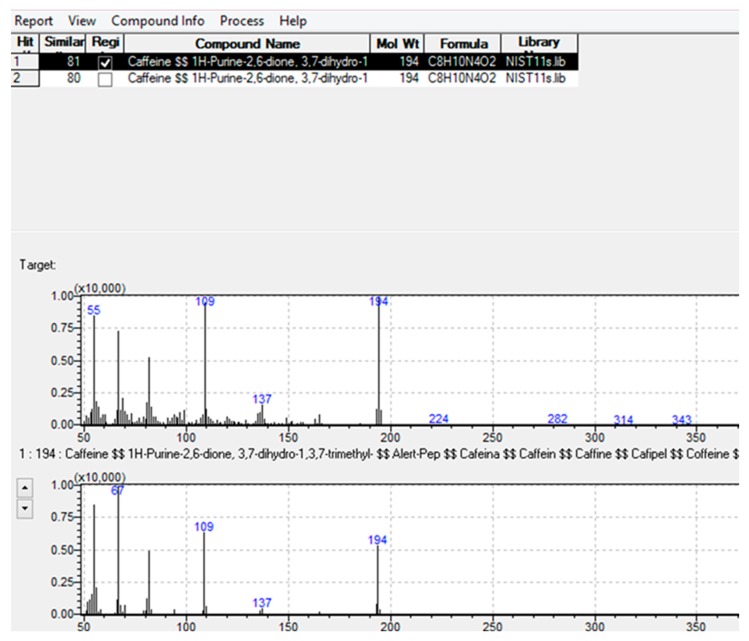
Comparison of measured caffeine spectra with NIST library spectra.

The GC-MS system was controlled using GCMS solution software (Version 4.11 SU1) from Shimadzu, Kyoto, Japan. The obtained data were analyzed using Postrun, which is an application manager of the GCMS solution. Postrun software allowed peak detection, assisted by automatic library searching, and similarity checks after integration. Integration is based on the peak area, height and rejection parameters, such as signal to noise ratio, slope, and drift. The instrument was operated in both full scan and single ion monitoring (SIM) mode for qualitative and quantitative analysis, respectively. Only analytes with an *m*/*z* between 30 and 850 were monitored and reported. The spectra measured by the instrument were matched with NIST (Version 11) library spectra based on these parameters: minimum similarity, search depth, hit number and retention index. The similarity index is a quantitative expression of the difference between the spectrum of an unknown sample and the spectrum recorded in a library as shown in Figure 2.

The similarity index (SI) is calculated using the equation below.

(1) $$\mathrm{SI}=\left(1-\frac{\sum_{\frac{m}{z}}∥\mathrm{lu}\left(\frac{m}{z}\right)-\mathrm{lt}\left(\frac{m}{z}\right)∥}{\sum_{\frac{m}{z}}\left\{\mathrm{lu}\left(\frac{m}{z}\right)+\mathrm{lt}\left(\frac{m}{z}\right)\right\}}\right)\times 100$$

lu(*m*/*z*): relative spectrum intensity of the *m*/*z* of the unknown mass spectrum.

lt(*m*/*z*): relative spectrum intensity of the *m*/*z* of a mass spectrum recorded in a library.

An SI of 100 indicates mass spectra that are identical, while an SI of 0 indicates spectra that are completely different as shown in Figure 2.

The search for a list of potential positive compounds was done by using the NIST database based on the trimethylsilyl (TMS) derivatives fragmentation pattern.

## 3. Confirmation of Standards

All target compounds were derivatized and injected into the GC-MS. The measured analytical information, such as retention time, fragmentation pattern and SIM ion, are presented in Table 1. This information was used to establish acceptable the similarity and rejection index of the methods as shown in Figure 2. The similarity index for caffeine was established to be 80% after injection of the caffeine standard and NIST library search was performed to identify the compound. The similarity index for most derivatized compounds matching with the spectra within the NIST library ranged from 60–100%, which was wide due to the added silylil group. The GC-MS results for selected compounds are included in the Supplementary Section.

This analytical extraction method was derived from our previous work, which was based on the analysis of acidic drugs and personal care products (PCPs) in both solid and water samples taken along the Mgeni and Msunduzi Rivers [1,24,25,26]. A number of studies have employed acetonitrile and methanol to extract various drug residues [27,28,29,30]. However, the acetone/ethyl acetate solvent system was preferred over methanol and acetonitrile because these solvents extracted a large number of matrix components, which complicated identification of known and unknown compounds in the samples [31,32]. The clean-up step played a major role when compounds with different physicochemical properties were extracted. An Oasis HLB cartridge was used over cyno and environmental cartridges because the Oasis HLB permitted adjustment of the pH over a wide range to retain different classes of compounds, which was not possible with the later cartridges. As a large number of emerging contaminants are polar and contain heteroatoms such as oxygen, the Oasis HLB cartridge captured a broad class of polar compounds besides the target contaminants. The recovery studies were performed with Oasis HLB, and the results are presented in Table 2. All the target analyte percentages were within the acceptable recoveries recommended by IUPAC [33]. The method detection limits were evaluated on river water, wastewater, biosolids and sediments spiked with target analytes. Spiking and extraction of samples was undertaken as described in the experimental section. Limit of detection (LOD) and limit of quantification (LOQ) for the target method were established by repeating the analysis 10 times at low concentration levels in four different matrices, and the results are presented in Table 2. Equations (2) and (3) were used to calculate LOD and LOQ, where σ is the standard deviation of the spiked sample, and s is the slope of the calibration curve [34].

(2) $$\mathrm{LOD}=\;\frac{\sigma }{s}\times 3$$

(3) $$\mathrm{LOQ}=\;\frac{\sigma }{s}\times 10$$

The LOD and LOQ values for river water and wastewater samples was higher than the sediment and biosolid limits. In addition, the Oasis cartridge absorbed more compounds, such as primary or secondary amines, which resulted in several of these compounds being detected [35]. Moreover, recovery of antibiotics was poor, and as a result, this class of compounds were not quantified in this study, except chloramphenicol.

## 4. Results and Discussion

### 4.1. Analysis of Environmental Samples: Target Analysis

The identification, confirmation and quantification of contaminants at trace level concentrations required a high sensitivity and selectivity to overcome a complex background matrix from biosolids and wastewater samples. Due to a large range of compounds targeted and a number of peaks detected in the environment, the GC-MS was operated in single ion monitored mode to get lower detection limits and improved selectivity.

#### 4.1.1. Quantification Analysis

A range of studies have shown that monitoring only one ion fragment might result in false positive identifications of contaminants, and in this work, two ions were selected to be monitored and used in quantification of target analytes [21,36,37,38,39]. In addition, the information provided in Table 1 was used to further confirm the presence of targeted analytes and eliminate errors in identification. In total, 12 compounds were targeted in four matrixes. Approximately 38 river water samples were collected and analyzed. Salicylic acid was below the quantification level in these rivers. Phenacetin and naproxen were found in higher concentrations in the Mgeni River as shown in Table 3. While triclosan and propylparaben were high in the Msunduzi River. In general, the Msunduzi River had a higher concentration of personal care products and the Mgeni River had a higher concentration of pharmaceuticals. Acetylsalicylic acid was found in higher concentrations in sediments in both rivers, followed by caffeine as presented in Table 3. Hence, salicylic acid, a metabolite of acetylsalicylic acid, was quantified in sediments in both rivers. Personal care products, as expected, were high in the Msunduzi River, as it is surrounded by informal settlements without proper sanitation. A number of WWTPs discharge their effluent to the Mgeni River and the concentration of pharmaceuticals, as expected, was higher than in the Msunduzi River. Environmental concentration levels obtained in this study were within range of the data from the literature [17,40,41,42].

Approximately 16 wastewater and biosolid samples collected from WWTPs in both municipalities were analyzed for target analysis. Caffeine was found to be dominant in wastewater samples from WWTPs in the Pietermaritzburg and Durban municipalities, and these results are presented in Table 4. All target analytes were quantified in biosolids. Diclofenac existed in high concentration with a median of 12 ng/g. Chloramphenicol, the only antibiotic quantified in the study, was found to range from ND to 16 ng/g, with a median of 5.3 ng/g, as shown in Table 4.

#### 4.1.2. Qualitative Analysis

Databases and libraries contain many spectral data for compounds, which can be used to qualitatively analyze samples. The compounds are listed in Table 5. Their standards were prepared, derivatized, and injected into the GC-MS. The fragmentation pattern and chromatic information is listed in Table 1. The R^2^ values for their calibration curves was below 0.9 and not satisfactory [33,34]. This was attributed to the difficulty in silylating amines due to steric hindrance. As a result, these compounds could not be quantified, and instead were qualitatively analyzed. Absolute recoveries were performed by spiking samples with 1 μg/L of these compounds. Percent recovery was calculated by subtracting blanks divided by 1 mg/L injected standard equivalent of 1 μg/L after extraction and pre-concentration. All recoveries ranged from 60–120%, limit of detection was taken as 1 μg/L for liquid samples and 10 ng/g for solid samples. Out of 14 analyzed, 13 were detected, and the only compound not found was procainamide as shown in Table 5. Most target analytes showed positive identification in biosolids compared to river water and wastewater samples.

### 4.2. Suspect Analysis

In contrast to target analysis, the suspects screening approach (see Table 6) did not rely on reference standards for identification of contaminants in samples. The peaks that were not target analytes, and eluted close to target analytes, were identified by using a NIST library search as described in Section 2.7.

With the help of the NIST library, measured spectra were matched and approximately 14 unknown peaks from the samples were identified as shown in Table 6. Among the identified peaks was the compound codeine, which is an active ingredient in cough syrup. This compound currently falls under the category of a drug of abuse in South Africa, because of its prevalence amongst illicit drug users. Clofibric acid, a metabolite of many lipid-based drugs, was detected in almost all matrices, specifically river water, wastewater and biosolids, and the results are presented in Table 6.

### 4.3. Non-Target Analysis

The compounds presented in Table 7 were analyzed without any prior knowledge of their existence in the environment. Because this study was undertaken to analyze PCPPs in the Msunduzi and Mgeni Rivers. The detected compounds in these rivers did not fall under the classification of PCPPs. Many of these contaminants were detected in wastewater and river water, more so than in biosolids and sediments as shown in Table 7. This was attributed to the fact that there were fewer interfering compounds in these matrices. Only a few compounds were identified in biosolids and sediments, although several peaks were detected in these matrices. This was attributed to the complexity of the biosolid and sediment matrices, which hindered the identification of compounds without prior knowledge of spectral behavior and other parameters such as retention indexes. The classes of these non-target compounds were hormones, paint, plasticizers, UV filters and flame retardants. There is a scarcity of information on the presence of these types of compounds in this part of the world. Detection of these contaminants will serve as motivation to prioritize further work on detection and quantification of hormones, flame retardants and plasticizers in the African environment at large.

## 5. Conclusions

Abundant fragments of silylated polar compounds were identified and used in GC-MS single ion monitoring mode to improve the sensitivity towards polar compounds in environmentally relevant concentrations. Obtained detection limits and recoveries allowed identification of these polar compounds in four compartments. Approximately 50 compounds were identified in river water, wastewater, sediment and biosolid samples collected from the Msunduzi and Mgeni Rivers. Out of the detected emerging contaminants, 12 were quantified mostly in sediments and biosolid matrices. Qualitative analysis was also performed and 14 compounds were found in the environment. Suspect and non-target analysis was also performed to identify unknown compounds using a readily available spectral library database, and 15 compounds previously not reported in the Msunduzi and Mgeni Rivers were found to exist. The use of GC-MS instrumentation and prior derivatization to identify up to 50 compounds successfully will serve as motivation to employ this method more often to study polar compounds in the environment. As GC-MS is more readily available in most laboratories in developing countries, more data will surface by using this method of detection.

## Figures and Tables

**Table 1:** Derivatized pharmaceutical and personal care GC-MS products obtained by injecting target standards.

Target Analytes TMS Derivative	Type	Chemical Formula Trimethylsilyl (TMS)	Retention Time Minutes	Fragment Pattern TMS Derivative (*m*/*z*)	Selected Ion Monitored (*m*/*z*)	Similarity Index
Methamphetamine	Illicit drug	C_10_H_15_N	5.450	58, 91, 134, 148	58, 91	84
Salicylic acid	NSAID	C_7_H_6_O_3_	6.345	73, 135, 193, 209, 267	135, 267	82
Acetylsalicylic acid	NSAID	C_9_H_8_O_4_	6.467	65, 73, 120, 195, 210, 268	120, 195	95
Nalidixic acid	Antibiotic	C_12_H_12_N_2_O_3_	6.914	73, 116, 162, 180, 236, 301	180, 236	89
Ibuprofen	NSAID	C_13_H_18_O_2_	7.105	73, 117, 160, 191, 263, 278	117, 160	80
Propylparaben	Antifungal agent	C_10_H_12_O_3_	7.458	73, 116, 162, 180, 236, 301, 251	162, 236	80
Phenacetin	Analgesic	C_10_H_13_NO_2_	7.800	53, 109, 137, 179, 209	109, 179	96
Acetaminophen	NSAID	C_8_H_9_NO_2_	8.000	73, 106, 166, 181, 223	181, 223	88
Phenoxyphenol	Standard	C_12_H_10_O_2_	8.250	73, 122, 150, 185, 258	150, 258	91
Morphine	Opioid analgesic	C_17_H_19_NO_3_	8.450	75, 103, 119, 174, 204, 232, 285	232, 204	81
Caffeine	Stimulant	C_8_H_10_N_4_O_2_	8.912	109, 194	109, 194	81
Naproxen	NSAID	C_14_H_14_O_3_	10.685	173, 41, 185, 243, 302	185, 243	88
procaine	Anaesthetic	C_13_H_20_N_2_O_2_	10.950	58, 86, 164	58, 86	81
Triclosan	Disinfectant	C_12_H_7_Cl_3_O_2_	11.250	109, 185, 200	109, 200	95
Meclofenamic acid	NSAID	C_14_H_11_Cl_2_NO_2_	11.990	73, 152, 208, 223, 313, 180	223, 313	95
Ketoprofen	NSAID	C_16_H_14_O_3_	12.105	73, 105, 165, 179, 253, 282, 311	282, 311	88
Diclofenac	NSAID	C_14_H_11_Cl_2_NO_2_	12.806	73, 93, 151, 214, 277, 367	214, 367	91
Carbamazepine	Anticonvulsant	C_15_H_12_N_2_O	13.654	63, 96, 165, 193, 236	193, 236	88
Chloramphenicol	Antibiotic	C_11_H_12_Cl_2_N_2_O_5_	13.921	73, 93, 147, 208, 225, 361, 451	208, 225	82
Cocaine	Illicit drug	C_17_H_21_NO_4_	14.530	77, 82, 152, 182, 272, 303	82, 182	91
Procainamide	Transformation	C_11_H_26_NO_2_	15.450	85, 99, 192	86, 99	88
2-phenylindolizine	Metabolite	C_14_H_11_N	15.960	63, 96, 165, 193	165, 193	80
Sulfamethoxazole	Antibiotic	C_10_H_11_N_3_O_3_S	16.500	65, 92, 156, 189, 253	92, 156	91
Chlorpromazine	Antipsychotic	C_17_H_19_ClN_2_S	17.605	58, 214, 232, 272, 315	58, 214	94
Lactose	Metabolite	C_36_H_86_O_16_	18.560	73, 103, 147, 204, 243, 319, 521	204, 243	81
Sulfamethazine	Antibiotic	C_12_H_14_N_4_O_2_S	20.052	92, 108, 156, 213, 277	92, 213	95
Clozapine	Antipsychotic	C_18_H_19_ClN_4_	21.750	70, 99, 164, 192, 243, 268, 326	192, 243	93

**Table 2:** Percent recovery, limit of detection and limit of quantification of derivatized compounds after spiking samples and performed recoveries.

Target Analytes	%Recovery				Limit of Detection (LOD)				Limit of Quantification (LOQ)			
	River Water %	Wastewater %	Sediments %	Biosolids %	River Water ng/L	Wastewater ng/L	Sediments ng/g	Biosolids ng/g	River Water ng/L	Wastewater ng/L	Sediments ng/g	Biosolids ng/g
Salicylic acid	70	65	100	105	41	51	0.04	0.17	135	164	0.15	0.56
Acetylsalicylic acid	99	90	91	102	285	403	0.02	0.09	950	1333	0.07	0.03
Ibuprofen	99	96	92	102	143	160	0.05	0.03	477	533	0.16	0.08
Propylparaben	98	102	94	105	1000	1500	0.10	0.14	4000	3000	0.3	0.42
Phenacetin	105	115	120	98	345	432	0.08	0.18	1151	1444	0.26	0.59
Caffeine	96	104	92	107	100	400	0.35	0.1	300	1100	1.07	0.33
Naproxen	82	80	66	112	75	101	0.08	0.03	248	333	0.280	0.104
Triclosan	100	94	91	108	89	100	0.08	0.1	270	290	0.25	0.36
Meclofenamic acid	103	106	85	121	82	111	0.11	0.14	272	368	0.38	0.46
Diclofenac	90	93	103	98	484	559	0.09	0.55	1614	1864	0.31	1.8
Carbamazepine	95	80	91	66	140	200	0.11	1.0	290	650	0.32	3.4
Chloramphenicol	98	102	98	102	100	500	1.8	2.5	250	1400	5.5	7.6

**Table 3:** Quantification of targeted contaminants in the Mgeni and Msunduzi Rivers.

Target Analytes	Mgeni River				Msunduzi River			
	River Water (ng/L)		Sediments (ng/g)		River Aater (ng/L)		Sediments (ng/g)	
	No. of Samples = 24		No. of Samples = 48		No. of Samples = 14		No. of Samples = 28	
	Range	Median	Range	Median	Range	Median	Range	Median
Salicylic acid	ND–D	D	ND–40	1.4	ND	ND	ND–3.43	0.28
Acetylsalicylic acid	ND–1130	70	ND–200	32	ND–D	D	ND–163	8.0
Ibuprofen	ND–2570	3870	ND–13	2.3	ND–D	D	ND–1.3	0.50
Propylparaben	ND–D	D	ND–13	1.1	ND–22,000	7000	ND–31	5.0
Phenacetin	ND–68,300	2300	ND–0.32	0.15	ND–2170	10	ND–0.67	0.30
Caffeine	ND–D	D	ND–128	2.0	ND–15,000	4500	ND–89	1.9
Naproxen	ND–59,000	2300	ND–15	0.98	ND–2380	580	ND–D	D
Triclosan	ND–5000	2000	ND–79	3.3	ND–20,000	2500	ND–43	4.9
Meclofenamic acid	ND–23,800	4201	ND–4.0	0.98	ND	ND	ND–2.8	1.0
Diclofenac	ND–1010	370	ND–3.75	0.91	ND	ND	ND–8.0	1.9
Carbamazepine	ND–D	D	ND–12	1.3	ND–D	D	ND–4.7	2.0
Chloramphenicol	ND–D	D	ND–5.0	0.54	ND–D	D	ND–19	5.0

ND = not detected, D = detected.

**Table 4:** Quantification of target compounds in wastewater and biosolids at wastewater treatment plants.

Target Analytes	Wastewater Treatment Plants			
	Wastewater (ng/L)		Biosolids (ng/g)	
	No. of Samples = 16		No. of Samples = 16	
	Range	Median	Range	Median
Salicylic acid	ND–66,000	820	ND–55	2.3
Acetylsalicylic acid	ND	ND	ND–221	29
Ibuprofen	ND–17,600	3000	ND–27	2.5
Propylparaben	ND–12,000	6200	ND–28	4.0
Phenacetin	ND–19,500	10	ND–40	5.0
Caffeine	D–15,000	7000	ND–173	24
Naproxen	ND–D	D	ND–13	3.0
Triclosan	ND–30,000	5000	ND–3.2	0.94
Meclofenamic acid	ND–2380	580	ND–86	8.0
Diclofenac	ND–10,200	250	ND–206	12
Carbamazepine	ND–D	D	ND–5.5	1.1
Chloramphenicol	ND–D	D	ND–16	5.3

ND = not detected, D = detected.

**Table 5:** Qualitative analysis of silylated target compounds in the Msunduzi and Mgeni Rivers.

Target Analytes	Schymanski Assessment Level [43]	River Water	Wastewater	Sediments	Biosolids
Acetaminophen	1	Detected	Detected	Detected	Detected
Ketoprofen	1	Detected	Detected	Detected	Detected
Sulfamethoxazole	1	Detected	-	-	-
Nalidixic acid	1	Detected	Detected	-	-
Sulfamethazine	1	-	-	-	Detected
Chlorpromazine	1	-	-	-	Detected
Clozapine	1	-	-	-	Detected
Procaine	1	-	-	-	Detected
Cocaine	1	-	Detected	-	-
Methamphetamine	1	Detected	Detected	Detected	Detected
Morphine	1	Detected	Detected	Detected	Detected
2-phenylindolizine	1	-	Detected	-	Detected
Lactose	1	-	-	-	Detected
Procainamide	1	-	-	-	-

**Table 6:** Suspect analysis of pharmaceutical and personal care products in the Msunduzi and Mgeni Rivers.

Suspect Analytes	Schymanski Assessment Level [43]	Chemical Formula	Fragment Pattern *m*/*z*	River Water	Wastewater	Sediments	Biosolids	Similarity Index
Clofibric acid	2	C_10_H_11_ClO_3_	39, 99, 128, 130, 214	Detected	Detected	-	Detected	75
Codeine	2	C_18_H_21_NO_3_	115, 162, 214, 229, 299	-	Detected	-	-	80
Oxazepam	2	C_15_H_11_ClN_2_O_2_	77, 205, 233, 239, 268	-	Detected	-	-	76
Trimethoprim	1	C_14_H_18_N_4_O_3_	123, 200, 243, 259,	-	-	-	Detected	91
Nicotine	2	C_10_H_14_N_2_	42, 84, 161	-	-	-	Detected	75
Amphetamine	1	C_9_H_13_N	44, 65, 91, 120	-	Detected	-	-	95
Benzoylecgonine	1	C_16_H_19_NO_4_	77, 82, 94, 124, 138,	-	Detected	-	-	95
Benzocaine	2	C_16_H_11_NO_2_	65, 92, 120, 137, 165	-	-	Detected	-	80
Cotinine	2	C_16_H_19_NO_4_	98, 176	-	-	-	-	79
Propranolol	2	C_16_H_21_NO_2_	30, 72, 115, 144, 331	Detected	-	-	-	80
Azelaic acid	2	C_9_H_14_Cl_2_O_2_	55, 83, 124, 152, 367	Detected	-	-	-	82
4-Oxoisophorone	2	C_9_H_12_O_2_	39, 68, 96, 152	-	Detected	-	-	80
Musk xylene	2	C_12_H_15_N_3_O_6_	43, 282	-	-	Detected	-	80
2-Pyrrolidone	2	C_4_H_7_NO	73, 142, 157	-		Detected	-	81
2-Phenoxyethanol	2	C_8_H_10_O_2_	77, 94, 138	Detected		-	-	79

**Table 7:** Non-target analysis of new emerging contaminants in the Msunduzi and Mgeni River at Schymansaki assessment level 3.

Non-Target Analytes	Source or Origin	Chemical Formula	Fragment Pattern *m*/*z*	River Water	Wastewater	Sediments	Biosolids	Similarity Index
Butyldiglycol	Paints	C_8_H_18_O_3_	57, 100, 132	Detected	Detected	-	-	79
2-propanol, 1-[1-methyl-2-(properyloxy)ether	-	C_9_H_18_O_3_	59, 103, 174	detected	-	-	-	80
Nicotinic acid	Vitamin	C_6_H_5_NO_2_	51, 91, 136, 195	-	Detected	-	-	72
Phenylmalonic acid	-	C_9_H_8_O_4_	69, 91, 136	-			-	88
2-ethyl-3-hydroxyhexyl 2-methyl propanoate	-	C_12_H_24_O_3_	71, 95, 99, 143, 174	Detected	Detected	-	-	79
Oxindole	Human metabolite	C_8_H_7_NO	78, 104, 133	Detected	Detected	-	-	81
2,6-Dimethylphenyl isocyanide	Cyanobacteria	C_9_H_9_NO	51, 118, 147	Detected	Detected	-	-	60
Obtusifoliol	Hormone	C_30_H_50_O	75, 215, 355, 370, 429	-	Detected	-	-	75
Cholesterol	Hormone	C_27_H_46_O	129, 329, 353, 368, 458	-	Detected	-	-	88
Metolachlor	Herbicide, pesticide	C_15_H_22_ClNO_2_	91, 162, 238	Detected		-	-	95
Bisphenol A	Plasticizer	C_15_H_16_O_2_	119, 213, 228, 372	-	-	-	-	96
Triethyl phosphate	Plasticizer	C_6_H_15_O_4_P	81, 99, 109, 155, 182	Detected	Detected	Detected	Detected	81
Triethyl citrate	Plasticizer	C_12_H_20_O_7_	115, 157, 203, 348	Detected	Detected	Detected	Detected	82
Oxybenzone	UV filters	C_14_H_12_O_3_	51, 77, 151, 227, 300		Detected	-	Detected	80
Tris(2-chloroethyl) phosphate	Flame retardant	C_6_H_12_Cl_3_O_4_P	63, 143, 205, 249, 253	Detected	-	-	-	79
Triphenyl phosphate	Flame retardant	C_18_H_15_O_4_P	77,169, 233, 233, 326	-	-	-	Detected	90

## Supplementary Materials

The following are available online at https://www.mdpi.com/1660-4601/16/20/4022/s1, Figure S1: Spectrum of derivatized chlorobenzoic acid. Obtained by injecting 2 μL of standard solution into GC-MS after derivatization, Figure S2: Spectrum of derivatized cinnamic acid. Obtained by injecting 2 μL of standard solution into GC-MS after derivatization, Figure S3: Spectrum of derivatized 4-phenoxyphenol. Obtained by injecting 2 μL of standard solution into GC-MS after derivatization, Figure S4: Spectrum of derivatized acetylsalicylic acid. Obtained by injecting 2 μL of standard solution into GC-MS after derivatization, Figure S5: Spectrum of derivatized ibuprofen. Obtained by injecting 2 μL of standard solution into GC-MS after derivatization, Figure S6: Spectrum of phenacetin. Obtained by injecting 2 μL of standard solution into GC-MS after derivatization. The lack of the silyl group indicated that this compound was not be derivatized, Figure S7: Spectrum of derivatized acetaminophen. Obtained by injecting 2 μL of standard solution into GC-MS after derivatization, Figure S8: Spectrum of derivatized acetaminophen. Obtained by injecting 2 μL of standard solution into GC-MS after derivatization, Figure S9: Spectrum of caffeine. Obtained by injecting 2 μL of standard solution into GC-MS after derivatization. The lack of the silyl group indicated that this compound was not be derivatized, Figure S10: Spectrum of derivatized carbamazepine. Obtained by injecting 2 μL of standard solution into GC-MS after derivatization. The lack of the silyl group indicated that this compound was not be derivatized, Figure S11: Spectrum of derivatized clozapine. Obtained by injecting 2 μL of standard solution into GC-MS after derivatization. The lack of the silyl group indicated that this compound was not be derivatized, Figure S12: Spectrum of derivatized chlorpromazine. Obtained by injecting 2 μL of standard solution into GC-MS after derivatization. The lack of the silyl group indicated that this compound was not be derivatized, Figure S13: Spectrum of sulfamethoxazole. Obtained by injecting 2 μL of standard solution into GC-MS after derivatization. The lack of the silyl group indicated that this compound was not be derivatized, Figure S14: Spectrum of sulfamethazine. Obtained by injecting 2 μL of standard solution into GC-MS after derivatization. The lack of the silyl group indicated that this compound was not be derivatized, Figure S15: Spectrum of derivatized chloramphenicol. Obtained by injecting 2 μL of standard solution into GC-MS after derivatization, Figure S16: Spectrum of cocaine. Obtained by injecting 2 μL of standard solution into GC-MS after derivatization. The lack of the silyl group indicated that this compound was not be derivatized, Figure S17: Spectrum of methamphetamine. Obtained by injecting 2 μL of standard solution into GC-MS after derivatization. The lack of the silyl group indicated that this compound was not be derivatized, Figure S18: Spectrum of morphine. Obtained by injecting 2 μL of standard solution into GC-MS after derivatization. The lack of the silyl group indicated that this compound was not be derivatized.
