# Supplementary material for: Target, Suspect and Non-Target Screening of Silylated Derivatives of Polar Compounds Based on Single Ion Monitoring GC-MS
Source: Int J Environ Res Public Health. 2019 Oct 21;16(20):4022. doi: 10.3390/ijerph16204022 (PMC6843951; doi:10.3390/ijerph16204022)
Supplement: Supplementary file 1 [file ijerph-16-04022-s001.pdf]

International Journal of
Environmental Research and Public Health

Supplementary Materials

Target, suspect and non-target screening of silylated derivatives of polar compounds based on single ion monitoring GC-MS

Chlorobenzoic acid

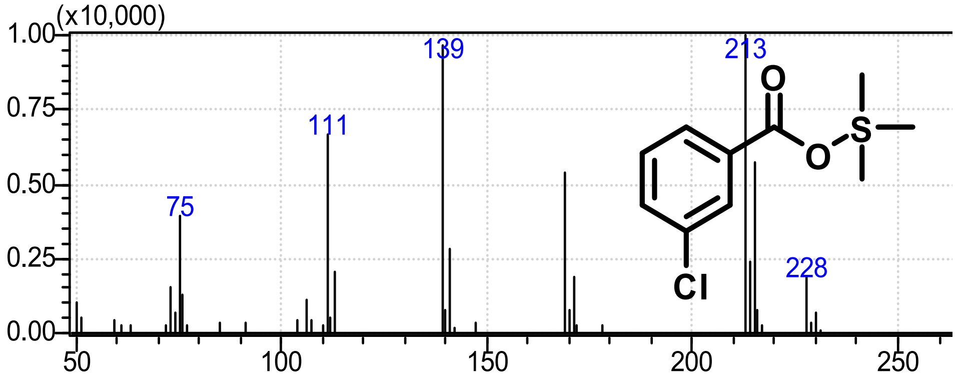

Figure S1. Spectrum of derivatized chlorobenzoic acid. Obtained by injecting 2 μL of standard solution into GC-MS after derivatization.

Cinnamic acid

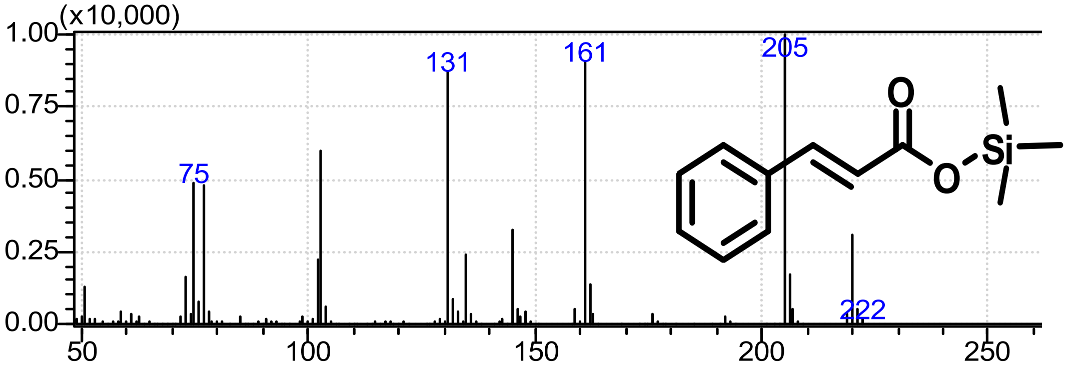

Figure S2. Spectrum of derivatized cinnamic acid. Obtained by injecting 2 μL of standard solution into GC-MS after derivatization.

4-Phenoxyphenol was used as an internal standard.

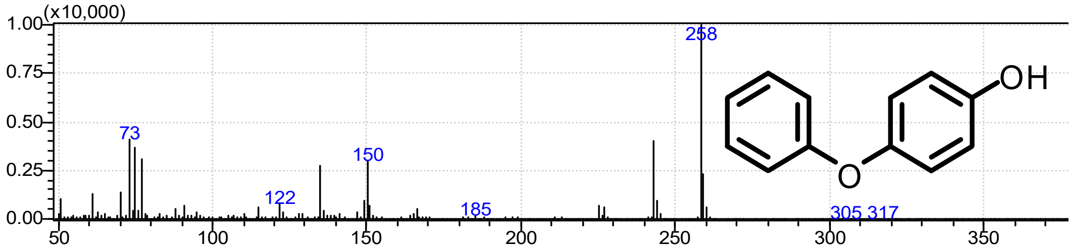

Figure S3. Spectrum of derivatized 4-phenoxyphenol. Obtained by injecting 2 μL of standard solution into GC-MS after derivatization.

Acetylsalicylic acid (aspirin)

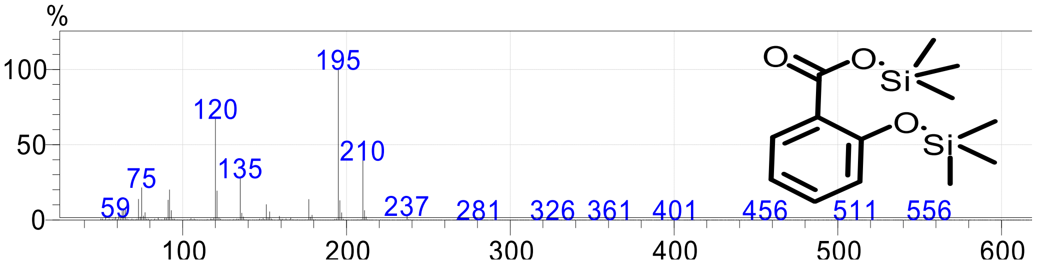

Figure S4. Spectrum of derivatized acetylsalicylic acid. Obtained by injecting 2 μL of standard solution into GC-MS after derivatization.

Ibuprofen

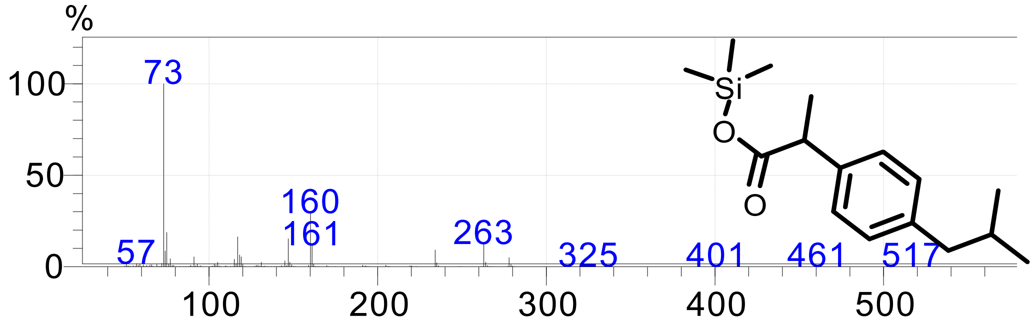

Figure S5. Spectrum of derivatized ibuprofen. Obtained by injecting 2 μL of standard solution into GC-MS after derivatization.

Phenacetin

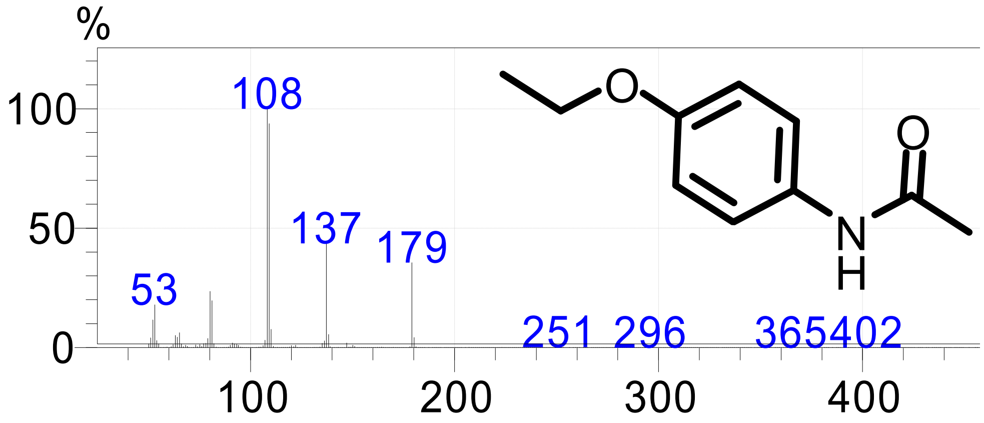

Figure S6. Spectrum of phenacetin. Obtained by injecting 2 μL of standard solution into GC-MS after derivatization. The lack of the silyl group indicated that this compound was not be derivatized.

Acetaminophen

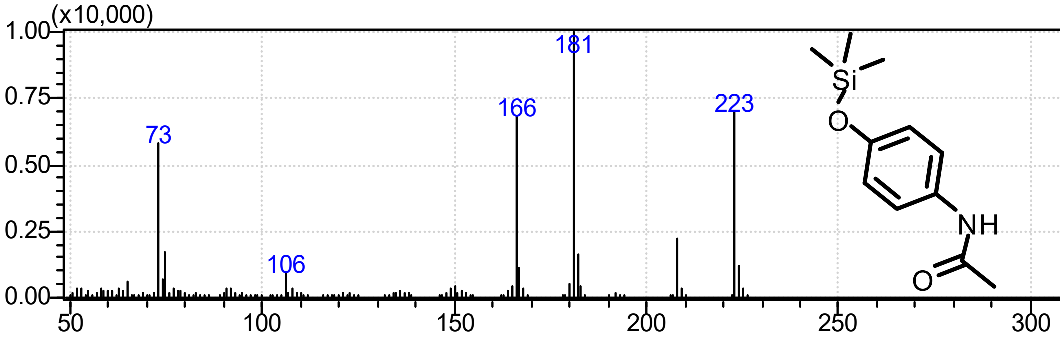

Figure S7. Spectrum of derivatized acetaminophen. Obtained by injecting 2 μL of standard solution into GC-MS after derivatization.

Naproxen

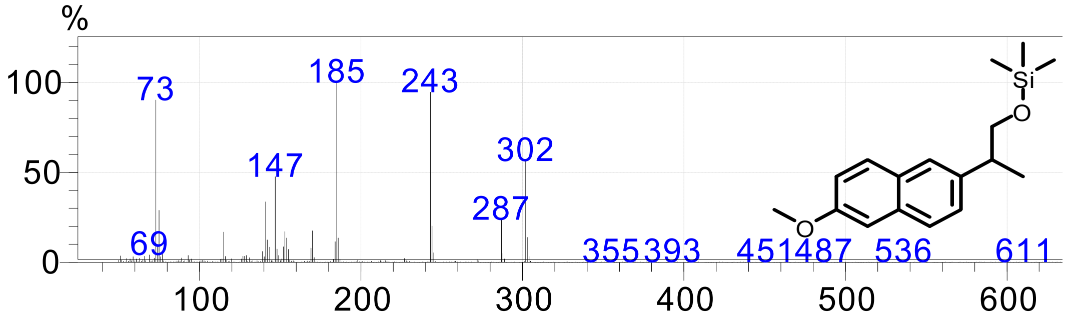

Figure S8. Spectrum of derivatized acetaminophen. Obtained by injecting 2 μL of standard solution into GC-MS after derivatization.

Caffeine

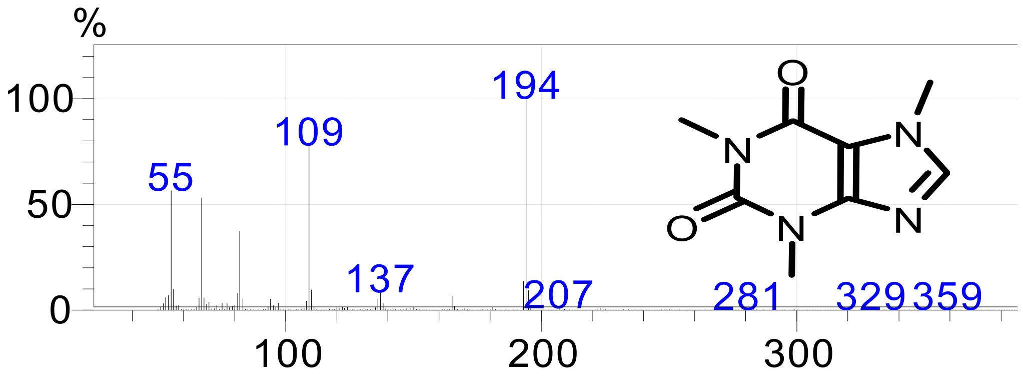

Figure S9. Spectrum of caffeine. Obtained by injecting 2 μL of standard solution into GC-MS after derivatization. The lack of the silyl group indicated that this compound was not be derivatized.

Carbamazepine

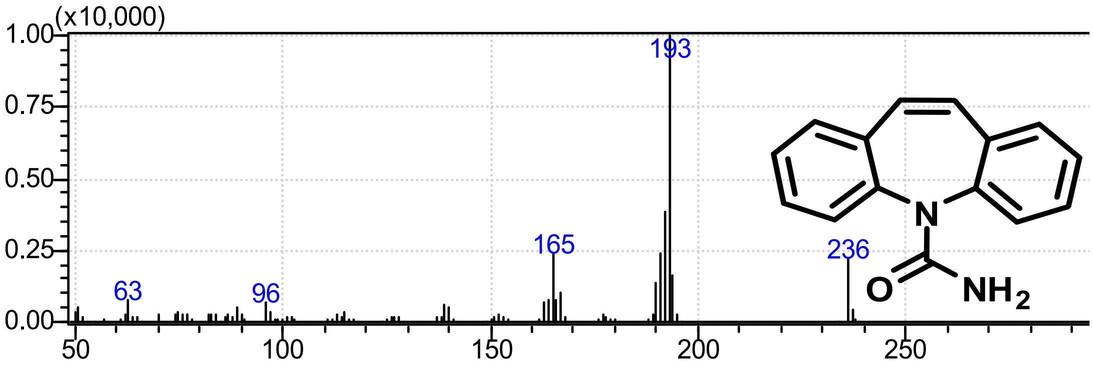

Figure S10: Spectrum of derivatized carbamazepine. Obtained by injecting 2 μL of standard solution into GC-MS after derivatization. The lack of the silyl group indicated that this compound was not be derivatized.

Clozapine

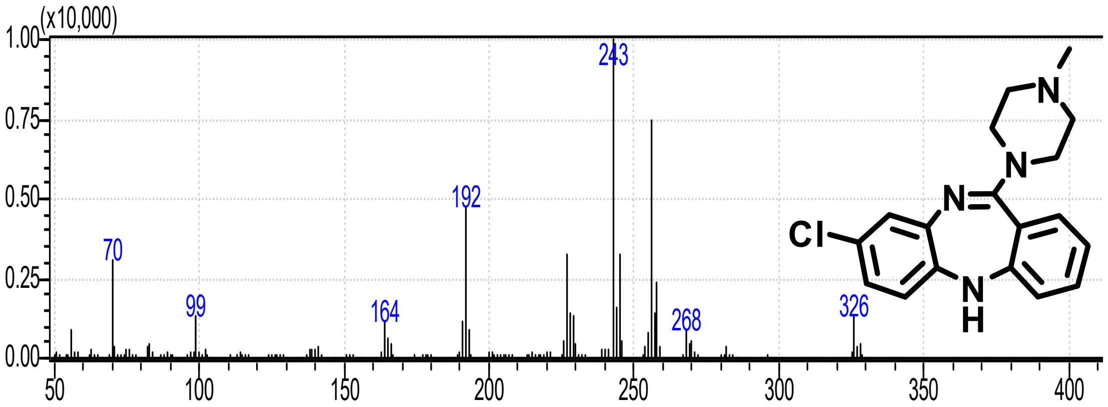

Figure S11. Spectrum of derivatized clozapine. Obtained by injecting 2 μL of standard solution into GC-MS after derivatization. The lack of the silyl group indicated that this compound was not be derivatized.

Chlorpromazine

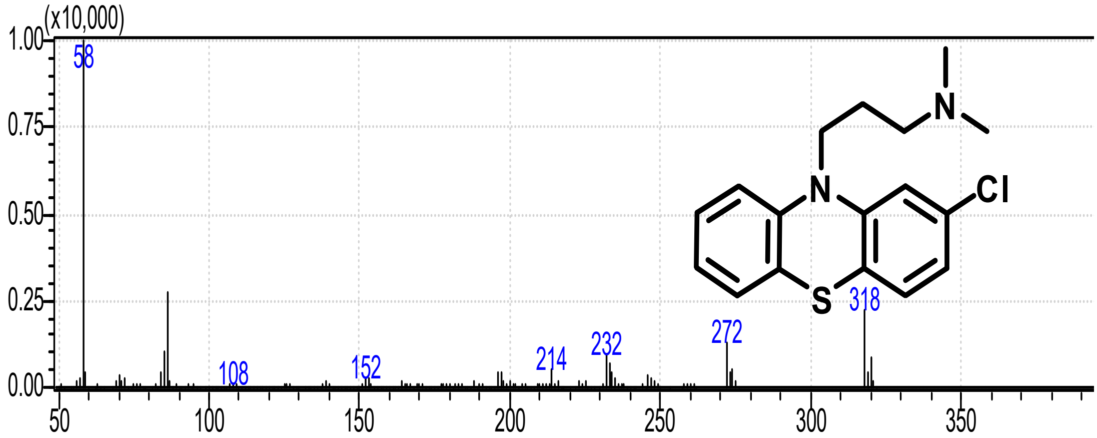

Figure S12. Spectrum of derivatized chlorpromazine. Obtained by injecting 2 μL of standard solution into GC-MS after derivatization. The lack of the silyl group indicated that this compound was not be derivatized.

Sulfamethoxazole

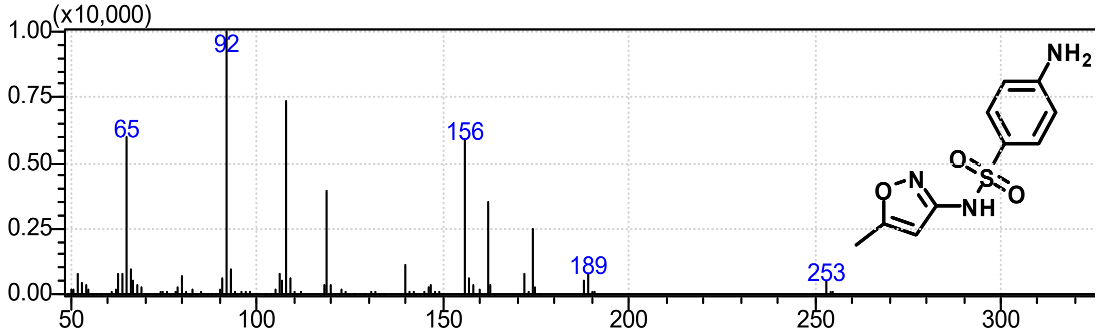

Figure S13. Spectrum of sulfamethoxazole. Obtained by injecting 2 μL of standard solution into GC-MS after derivatization. The lack of the silyl group indicated that this compound was not be derivatized.

Sulfamethazine

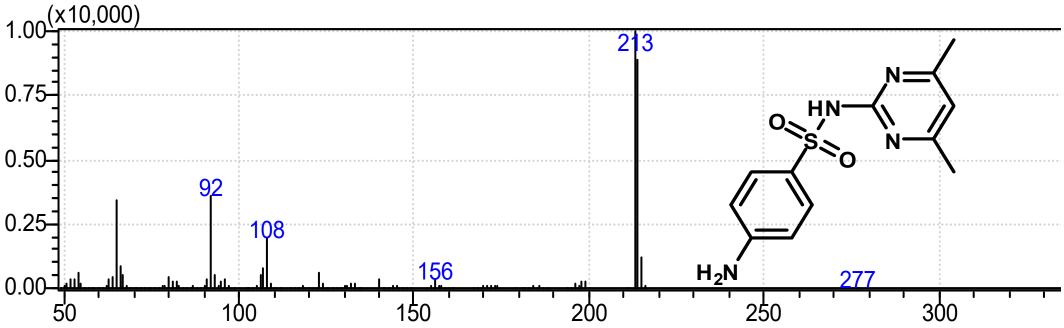

Figure S14. Spectrum of sulfamethazine. Obtained by injecting 2 μL of standard solution into GC-MS after derivatization. The lack of the silyl group indicated that this compound was not be derivatized.

Chloramphenicol

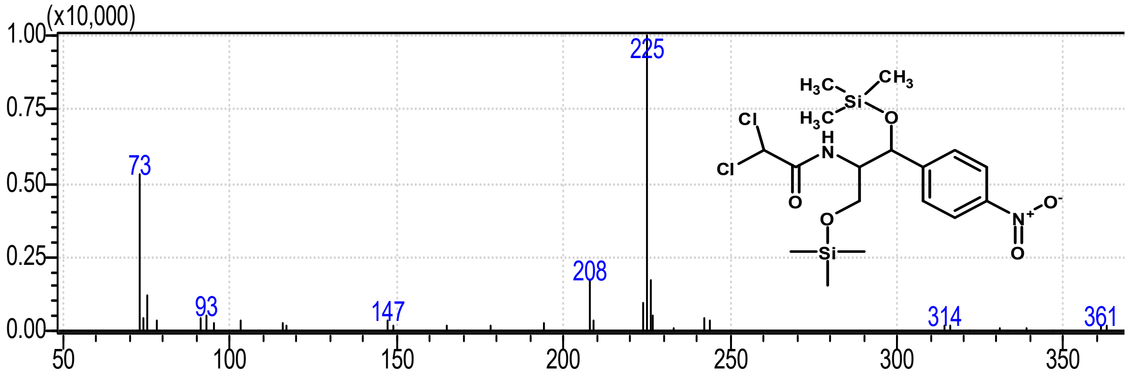

Figure S15. Spectrum of derivatized chloramphenicol. Obtained by injecting 2 μL of standard solution into GC-MS after derivatization.

Cocaine

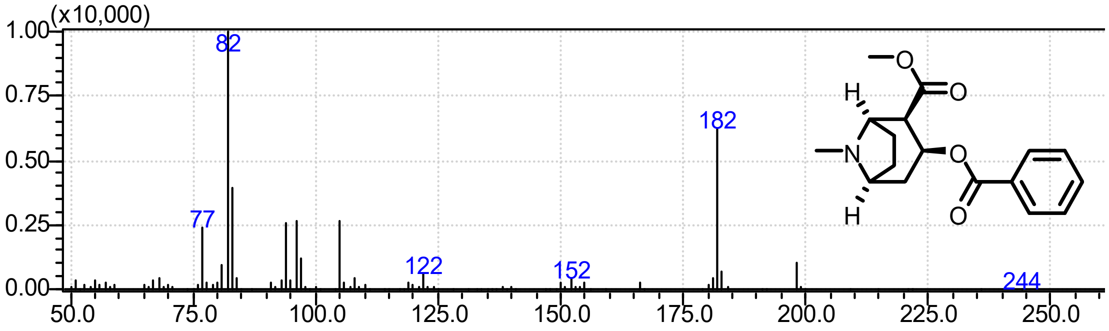

Figure S16. Spectrum of cocaine. Obtained by injecting 2 μL of standard solution into GC-MS after derivatization. The lack of the silyl group indicated that this compound was not be derivatized.

Methamphetamine

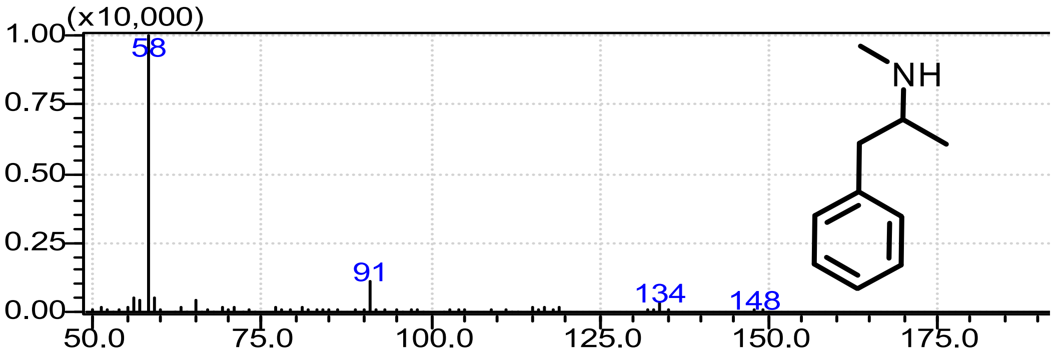

Figure S17. Spectrum of methamphetamine. Obtained by injecting 2 μL of standard solution into GC-MS after derivatization. The lack of the silyl group indicated that this compound was not be derivatized.

Morphine

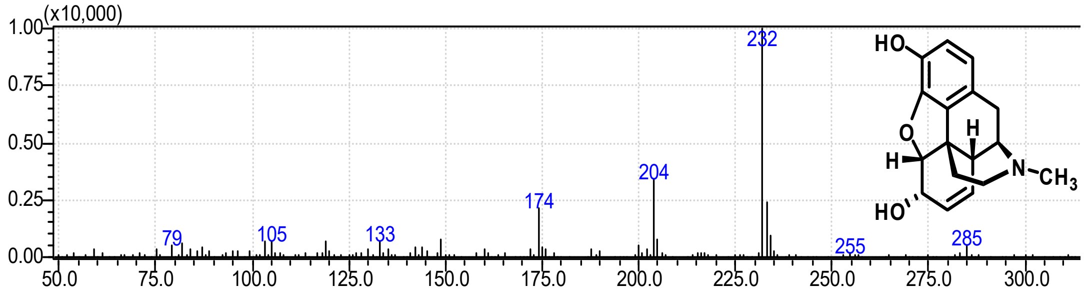

Figure S18. Spectrum of morphine. Obtained by injecting 2 μL of standard solution into GC-MS after derivatization. The lack of the silyl group indicated that this compound was not be derivatized.
